# Relationship between hsTnI and coronary stenosis in asymptomatic women with rheumatoid arthritis

**DOI:** 10.1186/s12872-016-0359-3

**Published:** 2016-09-29

**Authors:** Milan Hromádka, Jitka Seidlerová, Jan Baxa, David Suchý, Daniel Rajdl, Jakub Šedivý, Richard Rokyta

**Affiliations:** 1 Faculty of Medicine in Pilsen, Charles University, Plzen, Czech Republic; 2 Biomedical Centre, Faculty of Medicine in Pilsen, Charles University, Plzen, Czech Republic

**Keywords:** Calcium score, Coronary heart disease, Coronary CT angiography, High-sensitivity troponin, Rheumatoid arthritis

## Abstract

**Background:**

Rheumatoid arthritis (RA) is a condition associated with accelerated progression of atherosclerosis in affected individuals. Myocardial assessment using exercise testing in such patients, however, is often difficult to perform. Our objective was to determine the factors associated with severe coronary stenosis using computed tomography (CT) angiography of the coronary arteries in asymptomatic patients with RA.

**Methods:**

Forty-four women with RA were examined using CT angiography to detect atherosclerotic involvement and significant coronary stenosis (>50 %). CT findings were correlated with the cardiovascular risk score, and with classical and most recent parameters of atherosclerosis.

**Results:**

CT angiography of the coronary arteries revealed severe stenosis (>70 %) in 9 % of patients. High-sensitivity troponin I level was associated with severe coronary stenosis (odds ratio 6.37; 95 % confidence interval 1.53 − 26.48; *P* = 0.011). Adjustment for confounders did not alter this result (*P* = 0.039). In contrast, classical and modified Systemic Coronary Risk Evaluation scores had no value in predicting severe stenosis (*P* ≥ 0.49).

**Conclusion:**

The present study showed the possible benefits of a coronary CT angiography in women with RA and asymptomatic ischemic coronary heart disease. Increased levels of high-sensitivity troponin I may be a potential indication for this type of examination. However, further studies are needed to confirm these results.

## Background

Rheumatoid arthritis (RA) is an inflammatory autoimmune disease associated with a higher prevalence of cardiovascular (CV) disease, both of which are linked to the presence of traditional risk factors for atherosclerosis as well as chronic inflammation. Accelerated atherosclerosis of the coronary and cerebrovascular arteries is the main cause of morbidity and mortality in RA [1]. Patients with RA have a 45 % higher risk for all CV events than the general population, the risk for myocardial infarction is even higher (68 %) [2]. In addition, 30-day mortality after the first acute coronary event is 80 % higher in patients with RA than in the general population [3]. Early identification of high-risk patients and the initiation of preventive measures is, therefore, very important.

Classical CV Systematic Coronary Risk Evaluation (SCORE) may underestimate the actual risk in patients with RA, in whom inflammation plays a major role [4, 5]. Thus, the European League Against Rheumatism (EULAR) recommends multiplying the risk measured by SCORE by a factor of 1.5 in selected RA patients [6]. This applies to patients meeting 2 or more of the following criteria: duration of disease >10 years; positivity for rheumatoid factor or anti-citrulline antibodies; and the presence of certain extra-articular symptoms [6].

Currently, several imaging methods for the detection of coronary atherosclerotic lesions are available. Non-contrast CT is frequently used for assessment of calcium burden using Agatston calcium score [7]. Coronary CT angiography (cCTA) is, at present, used routinely in the diagnosis of coronary heart disease (CHD). This non-invasive method has high accuracy in detection of coronary stenosis and particularly predictive value in patients with negative findings [8–10].

The aim of the present study was to detect the following in asymptomatic patients with RA: the prevalence of significant coronary stenosis measured by cCTA; how coronary atherosclerosis diagnosed by CT examination correlates with classical or modified cardiovascular SCORE systems; and the predictive value of biomarkers in assessing the severity of coronary stenosis.

## Methods

### Study design

The prospectively designed single centre study included patients with RA who were at least 1 year on biological treatment observed in specialized outpatient clinic for rheumatological diseases during March and December 2013. Exclusion criteria were: 1) no other CV disease apart from arterial hypertension; 2) negative family medical history for diseases of the CV system; 3) current smoking; 4) symptoms suggesting the possibility of CHD; 5) contraindication to iodine contrast agent administration (allergy on iodine contrast agent and severe renal insufficiency). As RA is 3 times more frequent in women than in men, in those subjects eligible for this study, were only 2 men. To achieve higher homogeneity of our study we decide not to include them into present analysis. We further not included 1 patient because of allergy to iodine, 2 other women refused to participate.

All included patients were referred to cCTA and to take blood samples for laboratory assessment. Basic medical data, including co-morbidities, medications used, resting blood pressure, heart rate, height and weight were collected. Body mass index was calculated as body weight (kg)/height^2^ (m^2^). The stage of RA and the diagnosis of arterial hypertension, diabetes mellitus and dyslipidaemia were identified from medical documentation. The dose of corticosteroids used was recalculated as the equivalent dose of prednisone.

The CV SCORE (SCORE) and modified SCORE (mSCORE) were calculated in accordance with previously published recommendations [4, 6]. The CV SCORE takes into account patient’s sex, age, smoking status, systolic blood pressure and concentration of total cholesterol. Resulting number represents individual 10 year risk for fatal CV event. Modified SCORE multiplies the risk measured by SCORE by a factor of 1.5 in selected RA patients [6].

The Ethical Committee at the Faculty Hospital in Pilsen validated the study protocol, and all patients provided written informed consent. The study conformed to the provisions of the World Medical Association’s Declaration of Helsinki.

### Laboratory assessment

Standard biochemical parameters were assessed in the laboratory at the Faculty Hospital in Pilsen. The estimated glomerular filtration rate (eGFR) was calculated using the Modification of Diet in Renal Disease (MDRD) formula. Albuminuria is expressed as a urinary albumin-to-creatinine ratio (ACR). High-sensitivity cardiac troponin I (hsTnI) was measured using the Architect i2000 platform with STAT High Sensitive Troponin-I assay (Abbott Diagnostics, USA) (limit of quantification 4–10 ng/l, 10 % coefficient of variation 4.7 ng/l, 99th percentile 26.2 ng/l, percentage of measurable values in healthy population >80 %). Plasma levels of osteoprotegerin were measured using multiplex immunoanalyses based on xMAP® technology, commercially available kits MILLIPLEX MAP Human Bone Panel 1A magnetic (Merck-Millipore Corporation, USA) and the MAGPIX instrument (Luminex Corporation, USA). xMAP® technology and the MAGPIX system uses color-coded magnetic microspheres coated by antibodies to perform quantitative sandwich immunoanalysis of proteins in a variety of sample matrices.

### CT examination

#### Scanning parameters

CT examinations were performed using a second-generation dual-source CT device (SOMATOM Definition Flash, Siemens HealthCare, Forchheim, Germany). Initially, a non-contrast scan for calcium scoring quantification was performed. Cardiac CTA was monitored by retrospective electrocardiographic gating (128 × 0.6 mm; rotation 280 ms; adaptive pitch factor 0.2 − 0.4; optimized setting of tube voltage and tube current using CareDose and CarekV). Fifty millilitres of iodine contrast agent (400 mgI/l) was administered at a rate of 6 ml/s.

### Image analysis

Assessment of the examinations was performed in consensus by 2 radiologists experienced in CT heart examination (20 and 10 years, respectively). Calcium score was calculated using a dedicated Syngo. CaScoring application (Siemens Healthcare, Forchheim, Germany). The total calcium score, without differentiation of individual coronary arteries, was used for the analysis.

Coronary arteries were assessed according to the degree of luminal stenosis (mild: 10 % − 40 %; moderate: 40 % − 70 % and severe: >70 %). The significance of the degree of stenosis was assessed for each patient, and the characteristics of plaques were assessed according to individual segments [11].

### Statistical analysis

For statistical analysis, SAS software version 9.3 (SAS Institute Inc, USA) was used. The results are presented as arithmetic mean ± standard deviation, median with inter-quartile range (IQR) or as a proportion (percentage). For the purpose of statistical analysis, the subjects were divided into three groups according to maximal stenotic involvement of coronary arteries: without stenosis or mild stenosis according to cCTA (coronary stenosis <40 % [*n* = 28]); moderate stenosis (40 % − 70 % [*n* = 12]); and a group with severe stenosis (>70 % [*n* = 4]).

Differences among the groups were assessed using the paired Student’s *t* test, the Kruskal-Wallis test and the Fisher’s exact test. Correlations were expressed using the Spearman correlation coefficient. For the purposes of regression analysis, non-normally distributed variables were normalized using logarithmic transformation. Factors associated with a positive finding in CTAG were analysed using logistic regression. Significant attention was devoted to the selection of potential confounders. First, potentially significant covariables (age, arterial hypertension, dyslipidaemia, stage of RA, albuminuria, hs troponin I, dose of corticoids) were sought with a stepwise logistic regression analysis, which included only factors demonstrated to be significant in the univariate analysis. The final regression model included only parameters that remained in the models permanently.

## Results

### Patient characteristics

The mean age of 44 women included in present study was 60.2 years (range 50 − 70 years). Twenty-two women (50.0 %) were treated for arterial hypertension, 3 (6.8 %) had diabetes mellitus and 19 (43.2 %) had dyslipidaemia, of whom 16 were taking a statin.

The basic characteristics of the patients according to severity of coronary stenosis are summarized in Table 1. Arterial hypertension and dyslipidaemia were more prevalent in women with positive findings in the coronary arteries than in those with negative findings.

**Table 1:** Characteristics of patients according to severity of coronary stenosis

	Mild stenosis *n* = 28	Moderate stenosis *n* = 12	Severe stenosis, *n* = 4
Age	58.9 ± 5.0	63.6 ± 4.4**	58.7 ± 5.1
Systolic blood pressure, mm Hg	132.9 ± 10.8	127.1 ± 19.1	130.0 ± 8.2
Diastolic blood pressure, mm HG	80.9 ± 6.4	78.3 ± 6.15	87.5 ± 5.0
Arterial hypertension, n (%)	9 (32.1)	9 (75.0)*	4 (100.0) ^§^
Number of antihypertensive drugs, n	0 (0 – 1)	1 (0 -2)*	2 (1.5 – 2.5) ^§^
Heart rate, beat/min	70.8 ± 5.0	71.8 ± 6.7	76.5 ± 8.6
BMI, kg/m^2^	26.3 ± 3.8	28.3 ± 3.1	28.8 ± 2.2
Total cholesterol, mmol/l	5.22 ± 1.0	5.65 ± 0.88	5.96 ± 1.73
HDL cholesterol, mmol/l	1.92 ± 0.51	2.02 ± 0.70	2.06 ± 0.97
LDL cholesterol, mmol/l	2.86 ± 0.70	2.53 ± 1.20	3.82 ± 1.97
Triglycerides, mmol/l	1.41 ± 0.70	1.60 ± 0.63	1.99 ± 1.28
Apo-lipoprotein apoB, g/l	0.86 ± 0.17	0.91 ± 0.20	1.03 ± 0.34
Phospholipase A2, μg/l	127.2 ± 36.6	121.0 ± 24.1	104.5 ± 78.5
Serum creatinine, μmol/l	82.1 ± 13.3	81.2 ± 9.6	90.5 ± 28.1
eGFR, ml/s	1.06 ± 0.17	1.04 ± 0.15	0.99 ± 0.29
Dyslipidaemia, n (%)	8 (28.6)	8 (66.7)*	3 (75.0)
Treatment with statins, n (%)	7 (25.0)	7 (58.3)	2 (50.0)
Diabetes mellitus, n (%)	2 (7.1)	1 (8.3)	0
RA stage	2 (1 – 3)	3 (3 – 3)**	2.75 (2.25 -3.25)
FW, mm/h	20 (14 – 27)	26 (19 -46)	22 (16 - 26)
Corticoid dose, mg	2.1 (0 – 5.0)	4.5 (1.0 – 5.0)	6.9 (5.2 – 8.7) ^§^

The values are given as a number (percentage), mean ± standard deviation or median (interquartile difference)

*, **, ***P for the difference between the group with none to mild stenosis and moderate stenosis 0.05, 0.01, 0.001, respectively

§, §§, §§§*P* for the difference between the group with none to mild stenosis and severe stenosis 0.05, 0.01, 0.001, respectively

The median calcium score for the entire group was 21.5 (IQR 0 – 65.0). In our study, 21 (47.7 %) women had calcium score of 0; 15 (34.1 %) women had values between 1 and 100; and 8 (18.2 %) women had calcium score over 100. In all women with calcium score 0 we did not detected any atherosclerotic plaque. In the same line, 3 of 4 women with severe stenosis had calcium score higher than 100. Altogether 103 coronary segments with an atherosclerotic involvement were detected in 22 patients. Severe (>70 %) stenosis was observed in 4 patients, moderate stenosis in 12 patients and mild stenosis in 6 patients. The median number of coronary stenosis in the entire group was 1 (IQR 0 – 3.5) and there were distributed as follows: right coronary artery - 42 (40.8 %) lesions; left anterior descending artery - 37 (35.9 %) lesions; left circumflex artery - 24 (23.3 %) lesions. We found no lesion in left main artery. Only in 2 (9.1 %) patients we detected >40 % stenosis in 2 branches, the others had single vessel affected. Table 2 summarizes the values of biochemical parameters, CV risk SCORE and parameters of coronary atherosclerosis. Women with severe coronary stenosis exhibited significantly more severe micro-albuminuria, and higher hsTn1 and osteoprotegerin levels. As expected, the severity of coronary stenosis correlated with the calcium score and the number of coronary lesions.

**Table 2:** Biochemical parameters, CV risk SCORE and number of coronary lesions

	Mild stenosis, *n* = 28	Moderate stenosis *n* = 12	Severe stenosis, *n* = 4
ACR, mg/mmol	3.0 (0.9 – 3.0)	3.0 (2.0 – 3.0)	25.3 (21.5 – 29.2)^§^
hs troponin I, ng/l	2.05 (1.45 – 2.85)	2.90 (2.45 – 5.05)*	13.5 (6.4 – 21.0)^§§^
C reactive protein, mg/l	5.0 (2.0 – 7.0)	3.5 (2.0 – 8.0)	11.5 (3.5 – 21.0)
Osteoprotegerin, pg/ml	287 (230 – 397)	449 (381 – 655)**	565 (424 – 627)^§^
SCORE	1.5 (1.0 – 2.0)	4.0 (2.5 – 5.0)**	3.0 (1.0 – 5.5)
mSCORE	1.7 (1.0 – 3.0)	6.0 (3.0 – 7.2)**	4.0 (1.0 – 6.5)
Calcium score	0 (0 -13)	75 (39 – 185)***	319 (99.5 – 906)^§§^
Number of coronary lesions, n	0 (0 – 13)	3 (2 – 6)***	8.5 (6.0 – 11.0)^§§^
Number of calcified lesions, n	0 (0 – 0.5)	2 (2 – 5)***	6.5 (3.5 – 10.0)^§§^
Number of mixed lesions, n	0 (0 – 0.5)	0 (0 – 1)**	1 (0.5 – 1.5)^§§§^
Number of non-calcified lesions, n	0 (0 – 0)	0 (0 – 0)**	1 (0.5 – 1.0)^§§§^

For further explanation, see Table 1

*, **, ***P for the difference between the group with none to mild stenosis and moderate stenosis 0.05, 0.01, 0.001, respectively

§, §§, §§§ *P* for the difference between the group with none to mild stenosis and severe stenosis 0.05, 0.01, 0.001, respectively

**Fig. 1:**
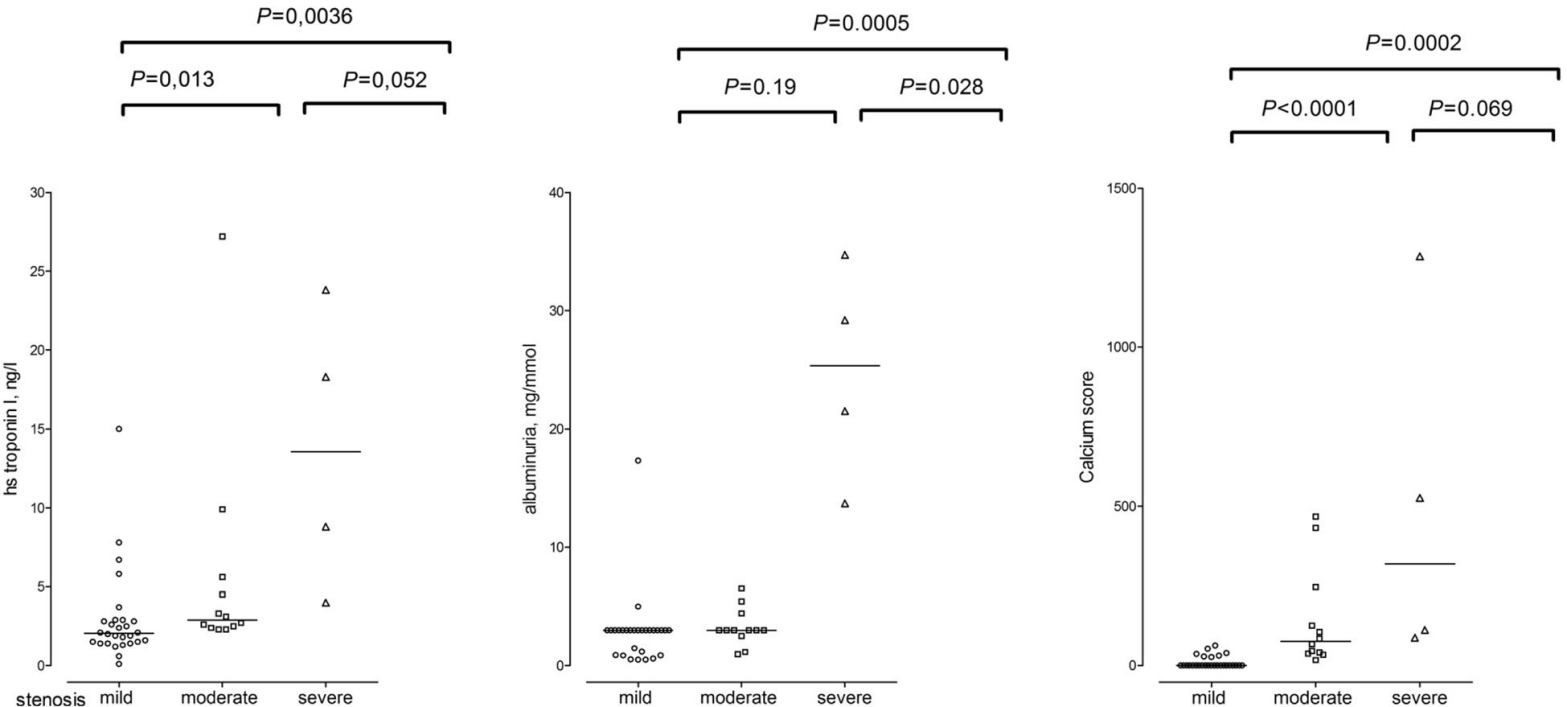
Values of high-sensitivity troponin I, albuminuria and calcium score according to the severity of coronary stenosis. Individual values and a median are shown. *P* for the difference between groups was calculated using the Kruskal-Wallis test

Figure 1 illustrates the hsTnl values, micro-albuminuria and calcium scores among the groups according to the severity of coronary stenosis.

Patients with severe coronary stenosis on cCTA were referred to coronary angiography. The findings were confirmatory to cCTA. On the basis of subsequent cardiologic examination, 2 patients were referred to coronary artery bypass surgery. Undergoing percutaneous coronary intervention was recommended to the remaining 2 patients to mitigate the risk of angina pectoris symptoms in the future.

### Factors associated with severe coronary artery stenosis using logistic regression

The only parameter consistently associated with severe coronary stenosis was hsTnl (univariate analysis, odds ratio [OR] 6.37; 95 % confidence interval [CI] 1.53 – 26.48; *P* = 0.011). Albuminuria reached borderline statistical significance (OR 2.39; 95 % CI 0.99 – 5.75; *P* = 0.052). Plasma levels of osteoprotegerin were not associated with severe coronary stenosis (OR 3.6; 95 % CI 0.52 – 17.96; *P* = 0.22). After adjustment for albuminuria and osteoprotegerin level, the hsTnl value remained significantly associated with severe coronary stenosis assessed by cCTA (OR 5.39; 95 % CI 1.09 – 26.64; *P* = 0.039).

Table 3 shows the correlation coefficients between calculated cardiovascular risk, hsTnl, albuminuria and the calcium score, and the number of coronary lesions. It is evident that the classical SCORE and mSCORE for CV risk were less strongly correlated with the number of coronary lesions than hsTnl values. The presence of albuminuria was not correlated with the calcium score or the number of coronary lesions.

**Table 3:** Correlation between the values of CV risk, hsTnl, calcium SCORE and the number of coronary lesions

	Calcium score	Number of coronary lesions
SCORE	0.41	0.45
*P*	*0.0052*	*0.0024*
mSCORE	0.40	0.43
*P*	*0.0064*	*0.0035*
hsTnI	0.57	0.58
*P*	*<0.0001*	*<0.0001*
albuminuria	0.19	0.14
*P*	*0.24*	*0.36*
Calcium score		0.96
*P*		*<0.0001*

**Fig. 2:**
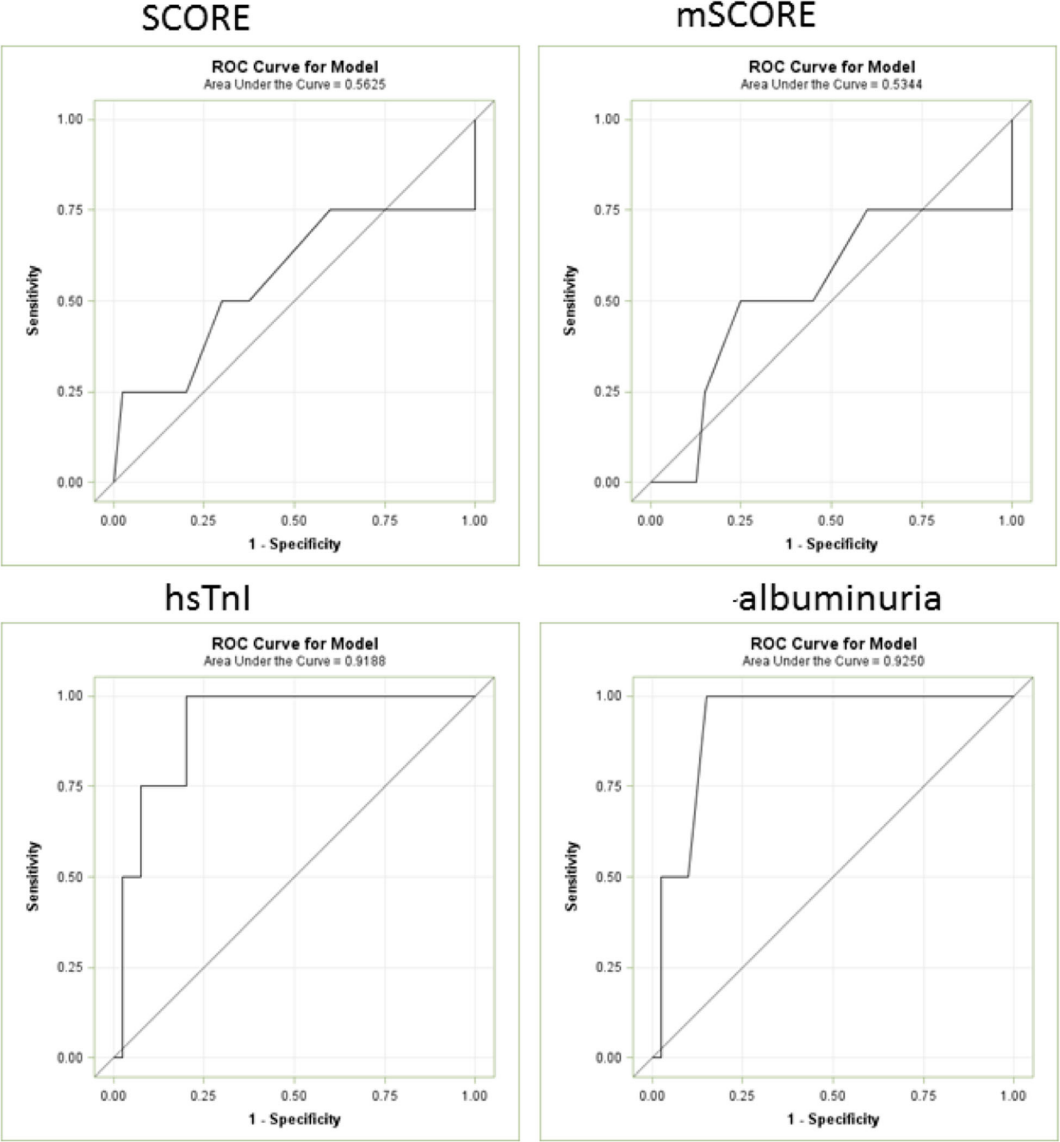
Receiver-operator characteristic curves for risk SCORE and biomarkers for predicting severe coronary stenosis

Figure 2 presents receiver-operator curves for classical SCORE and mSCORE, hsTnl and micro-albuminuria for the prediction of severe coronary stenosis. In this setting, SCORE (area under the curve [AUC] = 0.56) and mSCORE (AUC = 0.53) had no predictive value for severe stenosis. SCORE and mSCORE values in subjects with severe coronary stenosis fluctuated between 0 and 7. In contrast, hsTnl and albuminuria (ie, ACR) appeared to be biomarkers with very good sensitivity and specificity (AUC = 0.92 for both parameters).

## Discussion

In our study involving 44 women with rheumatoid arthritis without clinical symptoms of ischemic heart disease, we found that 9 % of them had severe coronary stenosis detected by coronary CT angiography. The classical cardiovascular risk SCORE or modified risk SCORE were not associated with coronary stenosis. On the other hand, high sensitivity Troponin I was correlated with the number and severity of coronary artery lesions.

RA is a disease associated with accelerated atherosclerosis and higher mortality after myocardial infarction in affected individuals [2, 3]. Early detection of significant CHD, thus, plays an important role in patient prognosis. Coronary CT angiography is non-invasive and safe method with possibility of excellent depiction of coronary plaques and stenosis assessment [12, 13].

High sensitivity cardiac troponins have demonstrated utility in estimating prognosis in general population and in patients with coronary heart disease, even in the range far below the 99th percentile (recommended cut-off value for diagnosis of acute myocardial infarction). deFillipi et al. [14] found a HR of 2.91 for cardiovascular mortality in older adults without known heart failure (>65 years; hsTnT > 12.94 ng/l vs < 3 ng/l). Omland et al. [15] (PEACE study) showed a HR 2.39 for cardiovascular mortality in stable CHD patients (hsTnT > 9.6 ng/l in men and > 7.4 ng/l in women) and Kavsak et al. [16] observed a HR 2.2 for cardiovascular mortality in stable patients at risk for CHD or chronic heart failure (hsTnI > 10 ng/l). Accordingly, hs troponins may be useful for risk stratification in patients with RA. In our study, hsTnl level had a high negative predictive value (18 of 21 patients without any coronary lesions had hsTnI < 3 ng/l).

Bradham et al. demonstrated that patients with RA had 49 % higher hsTnl levels than healthy individuals [17]. In the same study, the hsTnl value correlated with the calcium score in univariate analysis; however, after adjustment for age, race, sex and Framingham risk score, this association was no longer statistically significant. Our data suggest that female RA patients with the highest concentrations of hsTnI tend to have severe coronary stenosis (adjusted OR 5.39).

Of note, 99th percentile derived from our population of RA women without any signs of acute myocardial ischemia was 25.7 ng/l, whereas 99th percentile of the reference female population was 15 ng/l [18]. This fact should be probably considered in diagnostics of acute coronary syndromes in RA patients.

Moreover, we highlighted a potentially interesting connection between albuminuria and severe coronary stenosis. Albuminuria is a condition for which testing is readily available. Moreover, the presence of albuminuria increases CV risk by a factor of 2 − 4 [4]. Sihvonen et al. demonstrated that in patients with RA, the presence of albuminuria (in the range of 20–200 μg/min) increases the risk of mortality by 180 % compared with patients without simultaneously increased ACR [19]. In our study, albuminuria was correlated (borderline significance) with the severity of coronary stenosis, but not with the calcium score or the number of coronary lesions.

Other studies have demonstrated a connection between inflammatory markers and CV risk [1, 20, 21]. In our groups, however, we did not find any difference, either in erythrocyte sedimentation rate or C-reactive protein levels between women with positive and negative findings on cCTA.

Surprisingly, in our study classical or mSCORE systems did not correlate with the severity of coronary stenosis [4, 6]. On the other hand, calcium score enables to diagnose the severity of calcification in the coronary arteries. Increased calcium scores were detected in patients with RA [11] and were correlated with the occurrence of CV complications [22]. In our cohort, the calcium score was a very good predictor of severe coronary stenosis (AUC = 0.94). Moreover, it was significantly correlated (r = 0.96) with the total number of coronary lesions. Given its ready availability and its non-invasive character, cCTA is a suitable method for the detection of coronary atherosclerosis. A negative finding has a particularly strong predictive value with regard to stenotic lesions [23]. Coronary CTA is comparable with invasive angiography in its precision for assessing the degree of possible coronary arterial stenosis. However, its limitation lies mainly in its low specificity, which may overestimate stenosis, particularly in cases involving sclerotic plaque [23]. However, high sensitivity of cCTA is more important factor in our study and a negative cCTA finding has a strong predictive value with regard to stenotic lesions [24].

The present study had several limitations. First, it investigated a relatively small group of women with RA; our results, thus, cannot be generalized to include men or women without RA. However, our patients were comprehensively examined using a CT method that achieves excellent results in diagnosing CHD [25]. Second, the present study was cross-sectional in design; therefore, we have no long-term follow-up information regarding patient outcomes, although individuals with severe coronary stenosis have been shown to have a higher risk for major adverse cardiac events [26]. Third, the low prevalence of severe coronary stenosis in our group may lead to a skewed interpretation of the results, although we used several different statistical methods to analyse our data with confirmatory results. Four, we did not perform any stress test to assess functional severity of stenosis detected on cCTA. Moreover, we performed an invasive coronary angiography only in patients with finding of severe stenosis (<70 %). However, all patients were completely asymptomatic and we focused on non-invasive examination with minimized radiation and overall burden.

## Conclusion

Our study suggests the potential benefits of a coronary CT angiography in asymptomatic RA patients. In our hands, the hsTnl level was the best parameter for predicting of severe coronary stenosis assessed by CTAG. Classical or modified cardiovascular risk SCOREs were not effective in assessing the severity stenosis. However, further studies with larger patient cohorts and also correlation with clinical outcomes are needed before recommendation for measuring hsTnl levels in all patients with RA can be made.

## Abbreviations

ACR: Albumin-to-creatinine ratio
AUC: Area under curve
cCTA: Coronary CT angiography
CHD: Coronary heart disease
CT: Computed tomography
CTAG: Computed tomography angiography
CV: Cardiovascular
eGFR: Estimated glomerular filtration rate
EULAR: European league against rheumatism
hsTnI: High-sensitivity cardiac troponin I
MDRD: Modification of diet in renal disease
mSCORE: Modified systematic coronary risk evaluation
RA: Rheumatoid arthritis
SCORE: Systematic coronary risk evaluation
